# Cultural competency of health-care providers in a Swiss University Hospital: self-assessed cross-cultural skillfulness in a cross-sectional study

**DOI:** 10.1186/1472-6920-14-19

**Published:** 2014-01-30

**Authors:** Alejandra Casillas, Sophie Paroz, Alexander R Green, Hans Wolff, Orest Weber, Florence Faucherre, Françoise Ninane, Patrick Bodenmann

**Affiliations:** 1 Department of Ambulatory Care and Community Medicine, Lausanne University Hospital, Lausanne, Switzerland; 2 Department of Community Medicine and Public Health, Lausanne University Hospital, Lausanne, Switzerland; 3 Disparities Solutions Center, Massachusetts General Hospital, Harvard Medical School, Cambridge, USA; 4 Department of Primary Care, Community Medicine, and Emergencies, Geneva University Hospitals, Geneva, Switzerland; 5 Department of Psychiatry, Lausanne University Hospital, Lausanne, Switzerland

**Keywords:** Cultural competency, Cross-cultural care, Medical education, Health disparities, Vulnerable populations, Immigrant populations

## Abstract

**Background:**

As the diversity of the European population evolves, measuring providers’ skillfulness in cross-cultural care and understanding what contextual factors may influence this is increasingly necessary. Given limited information about differences in cultural competency by provider role, we compared cross-cultural skillfulness between physicians and nurses working at a Swiss university hospital.

**Methods:**

A survey on cross-cultural care was mailed in November 2010 to front-line providers in Lausanne, Switzerland. This questionnaire included some questions from the previously validated Cross-Cultural Care Survey. We compared physicians’ and nurses’ mean composite scores and proportion of “3-good/4-very good” responses, for nine perceived skillfulness items (4-point Likert-scale) using the validated tool. We used linear regression to examine how provider role (physician vs. nurse) was associated with composite skillfulness scores, adjusting for demographics (gender, non-French dominant language), workplace (time at institution, work-unit “sensitized” to cultural-care), reported cultural-competence training, and cross-cultural care problem-awareness.

**Results:**

Of 885 questionnaires, 368 (41.2%) returned the survey: 124 (33.6%) physicians and 244 (66.4%) nurses, reflecting institutional distribution of providers. Physicians had better mean composite scores for perceived skillfulness than nurses (2.7 vs. 2.5, p < 0.005), and significantly higher proportion of “good/very good” responses for 4/9 items. After adjusting for explanatory variables, physicians remained more likely to have higher skillfulness (β = 0.13, p = 0.05). Among all, higher skillfulness was associated with perception/awareness of problems in the following areas: inadequate cross-cultural training (β = 0.14, p = 0.01) and lack of practical experience caring for diverse populations (β = 0.11, p = 0.04). In stratified analyses among physicians alone, having French as a dominant language (β = −0.34, p < 0.005) was negatively correlated with skillfulness.

**Conclusions:**

Overall, there is much room for cultural competency improvement among providers. These results support the need for cross-cultural skills training with an inter-professional focus on nurses, education that attunes provider awareness to the local issues in cross-cultural care, and increased diversity efforts in the work force, particularly among physicians.

## Background

In the last decade, socio-cultural disparities in health status and access to care have come to the forefront of health services research, system reform, and policy. In its 2002 report *Unequal Treatment: Confronting Racial/Ethnic Disparities in Health Care*[1], the US Institute of Medicine brought attention to this area and recommended that cross-cultural education become integrated in the training of all current health professionals. Indeed, the field of cultural competence has emerged as one strategy to address these health disparities [2-4]: by educating providers to develop patient-centered approaches which focus on the socio-cultural factors that can affect a patient’s ability to beneficially engage the health system [5].

All countries confronting the demands of global immigration are faced with the need for 1) health providers that are skilled in cross-cultural competency, and 2) a method of assessment. Though Switzerland has been a land of immigration for some time, the Swiss health care system has seen an influx of new and vulnerable populations from around the world in the previous 15–20 years [6,7]. This diverse patient population represents a challenge to health professionals. Many are not aware that socio-cultural tension and misunderstanding between patients and providers can lead to patient mistrust, dissatisfaction, decreased confidence in the medical system, and ultimately, poor health outcomes [2,3,8-10]. To address these gaps, Lausanne University Hospital sought funding from the countrywide initiative “Swiss Migrant Friendly Hospitals” (MFH) to raise provider awareness and provide additional training and educational opportunities related to diversity and its importance in high-quality health care [6]. In preparation for these MFH projects, a baseline survey of “front-line health providers” (resident-physicians, chief residents, and clinical nurses) from the university hospital was conducted, to ascertain ability to care for vulnerable populations.

However, evaluation of provider cultural competency remains challenging [11,12] with many measures assessing cultural competency simply in relation to race and ethnicity [13], even when the definition of culture is broader (i.e. sexual identity, religion, socioeconomic/class) [14,5]. We used portions of an innovative, internally consistent, and valid self-assessment tool that better captures competencies related to all aspects of culture- with specific regard to perceived skillfulness [14]. Equally important in the present investigation is the comparison between physicians and nurses. Although there is literature to document differences between medical specialties [11,15,16], there is a paucity of literature examining the cross-cultural competency of nurses and other non-physician health providers [17]; even when cultural competence encompasses a general set of competencies that should be evaluated among all health providers [18,19]. This is contextually relevant in Switzerland, where nurses are increasingly assuming clinical responsibilities for vulnerable patients [20], and driving advocacy efforts in cultural competency training [21-23].

Therefore, the specific aims of this study were to 1) evaluate cross-cultural skillfulness and significant predictors of skillfulness among providers, and specifically, 2) compare cross-cultural skillfulness between physicians and nurses.

## Methods

### Setting

We used data from a cross-sectional survey at Lausanne University Hospital, one of five academic medical centers in Switzerland. At this academic medical center, socio-cultural patient diversity is part of clinical practice. More than 20% of the inhabitants of Lausanne speak a language other than French as a native language [7]. About a third of the patient population is not of Swiss-nationality, and includes undocumented and recently arrived immigrants seeking services in precarious states of health (Lausanne University Hospital internal database, 2011 administrative records, *Axya* software). The center serves other high-risk and vulnerable populations- homeless patients, drug and alcohol addicts, high-frequency emergency department users, uninsured individuals and patients with mental health issues. In 2011, the ambulatory clinic alone had 33,097 consultations with patients considered to be vulnerable (“Rapport Annuel PMU 2011”, Annual report for the Policlinique Médicale Universitaire in Lausanne).

### Population and recruitment

Eleven clinical divisions agreed to survey invitation (representative of 10 of 13 departments). “Front-line” health providers, defined as residents, chief residents and clinical nurses were invited to take the survey, given that they have the highest direct involvement with patient care at the institution. A survey was mailed in November 2010 to these front-line health providers. A repeat electronic mailing was sent 4 weeks after to those who had not responded. The Institutional Review Board approval for human subjects was obtained for this study from the Lausanne University Hospital ethics committee (addendum to “protocol du recherche 217/10”).

### General survey instrument

The written, self-administered, mail-in survey was in French, the official language of the Vaud canton. The final 64-item questionnaire broadly covered clinical and training experiences about cultural competence. Some questions were taken from the Cross-Cultural Care Survey (CCCS), a validated questionnaire developed in 2003 for resident physicians in the United States, initially administered across seven medical disciplines [14,15]. Other questions were added to capture relevant cultural competence aspects of the local setting, and these were elaborated partly with Geneva University Hospital, where local experts were developing a similar survey.

The survey was extensively reviewed and revised for use among this population of clinicians, achieving face-validation with nurses, a nurse leader and physicians during pre-testing (10 people in pilot tests- half nurses, half physicians). Questions were finalized with cultural competence experts in Switzerland and in the United States. Pilot testing was done to test the general structure/flow of the questions and the time needed to complete the survey. Questions that had been formulated for the CCCS were not changed as these had been previously validated. Testing was done to ensure that translation had not altered the original question.

### Measures- dependent variable

Our primary outcome is self-perceived skillfulness: nine Likert-response items about providers’ self-assessed skills to provide cross-cultural care which cover common and concrete items like language, that many other tools do not [13,14]. All of the skillfulness questions in this survey come directly from the CCCS. We used this outcome given the defined link between skillfulness and provider knowledge, training, positive attitudes, and role-modeling [24,15], demonstrating the superiority of skillfulness over other cultural-competency measures.

The skillfulness response scale in the survey ranged from “1 = not at all skillful” to “4 = very skillful.” As done in prior analyses of these items [25], we dichotomized the scaled responses for skillfulness: scores of three or four indicated “any skillfulness”, while scores of one and two were grouped as the categorical reference for “no skillfulness”. We also analyzed skillfulness as a composite score, summing respondents’ answers for each of the nine items, with higher scores indicating a higher level of skillfulness [11].

### Measures- independent variable and covariates

Providers were asked to answer whether they were a resident physician, chief resident physician, certified nurse or a specialized nurse. We compared physicians versus nurses in these analyses. We examined the effect of provider role on skillfulness after adjusting for demographic and workplace factors, and training and attitudes/awareness items related to cross-cultural care [25,26,16,19].

Demographic covariates were gender and whether the respondent’s dominant language was French. Workplace factors included time at institution, and respondents’ departmental division: we categorized department divisions as sensitized to cultural-care (sensitized versus non-sensitized). Sensitized divisions were pre-defined by the documented existence of specialized resources/services for diverse and vulnerable populations or having cultural competency training within the department. This classification was based on a recent internal communication summarizing the institution’s resources for diversity (“Questionnaire for heads of departments, to describe qualifying criteria for the Swiss network of Migrant Friendly Hospitals”). Providers’ reported training experiences were included, as these are associated with provider skillfulness [25]. These four items were from the CCCS (specifically using training experiences that may be encountered in this institution) where respondents answered “yes” or “no”.

Five questions from the CCCS on cross-cultural care awareness evaluated attitudes regarding the perceived impact of cross-cultural care on patient care [25]. Response options were “1 = no problem,” “2 = small problem,” “3 = moderate problem,” and “4 = big problem”. We dichotomized each of the five items with “moderate” and “big” grouped together.

### Statistical analyses

Using Student’s t-tests, we compared physicians’ and nurses’ mean composite scores for the nine self-perceived skillfulness items and proportion of “3-good/4-very good” responses using Chi-square tests. Accounting for any cluster effects secondary to division units, we used linear regression to examine how provider role (physician vs. nurse) was associated with composite scores, adjusting for demographics (gender, non-French dominant language), workplace (time at institution, work-division “sensitized” to cultural-care), items on reported cultural-competence training, and on cross-cultural care problem-awareness. We subsequently stratified analyses to examine significant predictors of skillfulness when looking at physicians and nurses separately. We present results significant at the p = 0.05 level. Stata 12.0 was used for all analyses.

## Results

Out of 885 mailed questionnaires, 368 individuals (41.2%) returned it: 124 (33.6%) physicians and 244 (66.4%) nurses, reflecting the distribution of providers in the institution. In terms of other socio-demographic and workplace characteristics (Table 1a), physicians were more likely to be male and have worked at the institution for less than five years. Among all, 59.7% of providers were “sensitized,” originating from departments that were institutionally exposed to some form of care or training resource.

**Table 1:** Characteristics of providers, perceived skillfulness explanatory factors (n = 368)

	**All providers %**	**Physicians, n = 124%**	**Nurses, n = 244%**	**p-value**^**†**^
**a) Demographics and workplace**				
Male	22.4	43.9	11.3	**<0.005**
French is dominant language	84.2	79.7	86.6	0.09
Time at institution is 5 years or less	57.6	87.7	42.3	**<0.005**
Sensitized Department	59.7	61.3	58.8	0.65
**b) Training experiences related to cross-cultural care:**“Have you had a training experience in…”^*^				
How to work with an interpreter	17.4	32.3	9.9	**<0.005**
Medical And social care network and affiliated organizations, for immigrant patients	23.6	37.1	16.8	**<0.005**
Support for patients without insurance or papers	31.6	57.3	18.5	**<0.005**
Historical or Cultural information on a specific group	15.3	18.7	13.6	0.20
**c) Cross-cultural care problem awareness:**“How much of a problem is each of the following…”^**^				
Lack of practical experience caring for diverse populations	71.2	69.1	72.3	0.52
Lack of time to address cultural issues	34.8	28.5	38.0	0.07
Inadequate cross-cultural training	43.3	47.5	41.1	0.24
Lack of access to informed interpreters	45.5	56.6	39.8	**<0.005**
Lack of good role models for cross-cultural care in the hospital	41.7	52.1	36.5	**0.01**

^**†**^Statistical tests- Comparison of proportions: Chi-square.

^*^Responded “yes”; ^**^Responded “big” or “moderate” (versus “no/small problem”).

Table 1b presents items related to training experiences. Although the percentages of all providers who received this training was in general low, a significantly higher percentage of physicians versus nurses responded “yes” for three of the four items. This was similarly observed among items related to cross-cultural care problem-awareness (Table 1c). Compared to nurses, physicians more frequently pointed out that there was a lack of access to informed interpreters and a lack of good role models for cross-cultural care in the hospital.

Table 2 shows that physicians had better mean composite scores for perceived skillfulness than nurses (2.67 vs. 2.50, p < 0.005), and significantly higher proportion of “good/very good” responses for four individual skillfulness items: taking a social history (93.5% vs. 49.6%, p < 0.005), working effectively through a medical interpreter (68.5% vs. 48.7%, p < 0.005), negotiating with the patient about key aspects of the treatment plan (81.8% vs. 55.8%, p < 0.005), and assessing patient understanding of the cause of his/her illness (75.0% vs. 61.0%, p = 0.01).

**Table 2:** Cross-cultural care perceived skillfulness, composite and itemized outcomes between physicians and nurses

***“How skillful are you at each of the following as you deliver cross-cultural care…”***	**Physicians (n = 124)**	**Nurses (n = 244)**	p-value^*^
Mean Composite^**^	**2.67, SD 0.38**	**2.50, SD 0.51**	**<0.005**
	**95% CI (2.60-2.74)**	**95% CI (2.44-2.57)**	
**Skillfulness items**^**†**^	% that were “skillful” or “very skillful”		
*Taking a social history*	93.5	49.6	**<0.005**
*Identifying how well the patient can read or write in French*	52.8	62.0	0.09
*Identifying cultural customs that might affect clinical care*	35.5	40.0	0.40
*Identifying religious beliefs that might affect clinical care*	29.3	36.5	0.17
*Working effectively through a medical interpreter*	68.5	48.7	**<0.005**
*Identifying how a patient makes decisions with other family members*	59.0	56.7	0.70
*Negotiating with the patient about key aspects of the treatment plan*	81.8	55.8	**<0.005**
*Assessing patient understanding of the cause of his/her illness*	75.0	61.0	**0.01**
*Identifying the level of patient trust in the health care system*	43.8	48.5	0.39

^*^Statistical tests- Comparison of proportions: Chi-square, Test for mean composites: Independent Samples t-test.

^**^Likert scale of 1 to 4.

^†^Ref. % of 1-“not at all skillful” and 2-“not skillful”.

In bivariate analyses among all providers (data not shown for simple linear regressions), physician role was significantly associated with skillfulness (β = 0.17, p < 0.005), while French as a dominant language was associated with lower skillfulness (β = −0.16, p = 0.02). All items on provider training and problem-awareness were associated with increased skillfulness.

The effect of provider role- with higher skillfulness associated with physicians, remained significant even after completely adjusting for possible explanatory factors in the multivariate models (β = 0.13, p = 0.05) (Table 3). Among all, two problem-awareness items were significantly correlated with higher skillfulness: awareness about inadequate cross-cultural training (β = 0.14, p = 0.01) and awareness about the lack of practical experience available in caring for diverse populations (β = 0.11, p = 0.04).

**Table 3:** Multivariate linear regression models of perceived skillfulness composite

	**All providers**	**Physicians alone**	**Nurses alone**
**Predictors**	**β =**	**β =**	**β =**
**Main**			
Provider role	**0.13**		
(Ref. nurse)	**p = 0.05**		
**Demographic**			
Male	−0.02	0.04	−0.05
(Ref. female)	p = 0.76	p = 0.55	p = 0.06
French is dominant language	−0.11	**−0.34**	0.06
(Ref. non-French)	p = 0.09	**<0.005**	p = 0.56
**Workplace**			
Sensitized department	−0.02	−0.02	−0.01
(Ref. non-sensitized)	p = 0.74	p = 0.84	p = 0.94
Time at institution is 5 years or less	−0.00	−0.05	0.02
(Ref. more than 5 years)	p = 0.94	p = 0.56	p = 0.72
**Had specific training experiences** “*Have you had a training experience in…”* (Ref. “no”)			
*How to work with an interpreter*	0.09	0.10	0.21
	p = 0.26	p = 0.25	p = 0.098
*Medical and Social care network and affiliated organizations, for immigrant patients*	0.10	0.14	0.13
	p = 0.15	p = 0.12	p = 0.22
*Support for patients without insurance or papers*	−0.06	−0.10	−0.11
	p = 0.36	p = 0.22	p = 0.27
*Historical or Cultural information on a specific group*	0.10	−0.14	**0.25**
	p = 0.17	p = 0.15	**p = 0.02**
**Had problem awareness about issues in cross-cultural care** “*How much of a problem is…*” (Ref. “*not at all/a little*”)			
*Lack of practical experience caring for diverse populations*	**0.11**	0.08	**0.16**
	**p = 0.04**	p = 0.32	**p = 0.04**
*Lack of time to address cultural issues*	0.06	0.07	0.06
	p = 0.30	p = 0.39	p = 0.40
*Inadequate cross-cultural training*	**0.14**	**0.22**	0.08
	**p = 0.01**	**<0.005**	p = 0.32
*Lack of access to informed interpreters*	0.02	0.07	0.05
	p = 0.66	p = 0.36	p = 0.55
*Lack of good role models for cross-cultural care in the hospital*	0.04	0.01	0.05
	p = 0.47	p = 0.85	p = 0.55
Adjusted R^2^	0.16	0.34	0.13

In stratified multivariate analyses among physicians alone, having a French dominant language (β = −0.34, p < 0.005) was negatively associated with skillfulness, while problem-awareness about inadequate cross-cultural training (β = 0.22, p < 0.005), had a positive association. Among nurses alone, having received training on the history/culture of a specific group (β = 0.25, p = 0.02) was associated with skillfulness, as was problem-awareness about the lack of practical experience caring for diverse populations (β = 0.16, p = 0.04).

## Discussion

Overall, there is much room for improvement of cultural competency among Lausanne providers. These results support the need for cross-cultural skill training with an inter-professional focus, education that attunes provider awareness to the local issues in cross-cultural care, and improvement of diversity efforts in the work force, particularly among physicians.

Notably, provider role made a difference regarding skillfulness, even after adjustment. The lower perceived skillfulness scores among Swiss nurses was a surprising finding. The nursing profession in Switzerland is known for emphasizing the topic of cultural competency, with many nurse voices advocating for better cross-cultural care. However, nurses have commonly expressed difficulty in having their concerns/questions addressed in the treatment of vulnerable patients, on account of historical inequalities between nursing staff and physicians in Swiss health-centers [23]. Also in these analyses, we observed that nurses reported less access to training resources, compared to physicians- possibly also because of the historical inequalities between the two professions. The impact of provider role on cross-cultural skillfulness clearly signals the need for inter-professional health-education in Swiss cultural competency efforts, a collaborative movement that is already seeing momentum in the United States across different training areas in health care [27,28].

In this context, it is also possible that nurses rated themselves lower on skillfulness, because they are more self-aware or have higher expectations of themselves, compared to physicians. Physicians may be less likely to admit to a lack of competency (differential professional expectations). Although more research is needed to explain this discrepancy, this phenomenon has been observed before. In a study of Hawaiian general surgery and family practice residents, the surgical trainees tended to score higher on self-rated skillfulness compared to those in primary care, despite having less formal training on the topics [11]. This disconnect may be linked to cultural humility—more familiarity with cross-cultural care that paradoxically leads to feelings of being less prepared or skillful in this topic [11,14]. However, in addition to lower skillfulness, Swiss nurses also reported less formal training than physicians: placing attention on a training gap between front-line providers, which could explain their lower skillfulness.

Two items related to problem awareness about cross-cultural care were positively associated with skillfulness among all providers together: awareness about “lack of practical experience caring for diverse populations” and “inadequate cross-cultural training”. Surprisingly, specific training experience items did not factor in as strongly towards higher skillfulness. Perhaps this suggests that those who are already interested in, and aware of the issues in cross-cultural care, tend to be more skilled at baseline, and desire more training than is currently provided. It is possible that a heightened sensitivity to the local issues in cross-cultural care are reflective of individuals who have received a broad, in-depth skills training, that is not as well-captured by the individual training items that we utilized. We did not include all types of training, given power restrictions, but we did incorporate a selection of training experiences that reflect important issues in cross-cultural care, from a conceptual framework standpoint (language/interpreter navigation, network/system knowledge, social support resources, cultural knowledge) [2,8,10]. The results suggest that improving providers’ awareness about the local pertinent problems in cross-cultural care - as a specific topic in cross-cultural education and, also as a result of other training, may improve skillfulness.

In stratified models, significant explanatory factors differed between physicians and nurses, as expected. Notably, French, as a dominant language was associated with lower skillfulness among physicians. Our hypothesis is that a non-French dominant language in Lausanne reflects a foreign culture for the physician, and thus a heightened sensitivity to diverse patients. Other studies have found similar results- in one evaluation of American medical students’ self-rated skill to care for limited English proficient patients, student race/ethnicity was one of only two variables which remained significant in multivariate regression [29]. This reinforces the message that increased diversity efforts can be a mechanism for increasing cultural competency in the workforce.

Our study has several limitations. Because this was a secondary data analysis, we were limited by the amount and type of information, which had already been collected. Notably, we were not able to adjust the models for the effect of respondents’ age. In this case, we used “time at institution” as an alternative, acknowledging nevertheless that this is not a perfect substitute. Although the 41.2% response rate was lower than would be desired for a survey, the response was actually better than other studies done in this population type and setting [30] (“General response rates of surveys among providers at Lausanne University Hospital”, 2012 personal communication to Dr. Patrick Bodenmann by Professor Bernand Burnand). In further analyses, we found no significant differences between the final sample and the initial recruitment pool in terms of gender and percentage of physicians versus nurses. Because the study was limited to one institution, the results may not be readily generalizable. However, this setting is a large academic medical center facing stresses and tensions in caring for diverse and vulnerable patients, similar to the situation in other university medical centers in Switzerland and abroad.

The survey is a self-reported measure, reflecting the perceptions of the respondents, not measured skills. However, this limitation would be more concerning if self-ratings were unusually high, suggesting socially desirable responses. Because mean skillfulness composites were lower in this group than in previous studies [14,24], this potential limitation is less concerning. It is also worthwhile to note that self-assessments are “acknowledged as an important component of adult and lifelong learning, and have been used in previous studies of educational quality, and shown to be valid predictors of examination scores, and faculty evaluations” [26]. Nonetheless, new research should delve into measuring the correlation between patient ratings and self-assessment, in order to continually rely on these tools [3].

Another potential issue is that this is the first time that the CCCS has been utilized with nurses. One may question the validity of survey items in this context, especially when physicians and nurses experience different medical educations. There may be innate professional differences in the manner in which both groups respond, which we cannot readily adjust for. A baseline comparison of these measures is still relevant, as these items have been useful in comparing medical students and physicians from very different specialties and settings, with different education and training. The line items for the skillfulness tool reflect a general set of health care worker competencies that should be evaluated among all providers; as such, other literature has proposed the utility of using the CCCS for all types of health care providers in designing culturally competent practices [24,11].

## Conclusions

All patients will benefit from culturally-competent providers who take account of the patient personal context and acknowledge his/her way of life, in the development of treatment plans and care [21,22]. Health centers should continue to evaluate these competencies in all providers and settings- institutions should also 1) address the efficacy of current training programs in regards to measured skillfulness, 2) develop further explanatory factors for skillfulness, and 3) amend the current strategies in training to address discovered gaps between nurses and physicians. At Lausanne University Hospital, we plan to repeat a survey in 2015 to document progress in provider perceived competencies following the curricular changes we have made as part of the aforementioned Swiss MFH project. Our results support the notion that immediate efforts to improve skillfulness among providers can be enhanced by institutional mechanisms- specifically by increasing inter-professional education efforts, improving providers’ awareness about cross-cultural issues in the local environment, and by prioritizing recruitment strategies for health care workers from diverse backgrounds.

## Abbreviations

MFH: Migrant friendly hospitals; CCCS: Cross-cultural care survey.

## Competing interests

The authors declare that they have no competing interests.

## Authors’ contributions

AC conceived the analysis idea, planned the study design and carried out the statistical studies, and drafted the manuscript. SP developed and carried out the original survey, and edited revisions of the manuscript. AG participated in the development of the survey and review of the analysis plan and the manuscript. HW participated in the design of the study and review of the manuscript. OW, FF, and FN are part of a multidisciplinary team (psychiatry, medicine, and nursing) that participated in the development of the original survey and review of the manuscript. PB is the senior mentor who obtained funding for the original project, guided all analyses and supervised the writing of the manuscript. All authors read and approved the final manuscript.

## Pre-publication history

The pre-publication history for this paper can be accessed here:

http://www.biomedcentral.com/1472-6920/14/19/prepub

## Survey note

We are happy to make a copy of the survey used available to anyone who is interested.
